# 离子色谱在中草药成分分析中的应用

**DOI:** 10.3724/SP.J.1123.2023.10009

**Published:** 2024-04-08

**Authors:** Baoxin ZHANG, Jingqin TIAN, Guozhu CHAI, Wenqi HE, Xiaozhong LAN, Xinghao HAN

**Affiliations:** 1.西藏大学医学院, 西藏 拉萨 850000; 1. Medicine College, Tibet University, Lhasa 850000, China; 2.西藏农牧学院, 西藏中(藏)药资源中心, 西藏自治区藏药资源保护与利用重点实验室, 西藏 林芝 860000; 2. Key Laboratory of Tibetan Medicine Resources Conservation and Utilization of Tibet Autonomous Region, Tibet Chinese (Tibetan) Medicine Resources Center, Tibet Agriculture and Animal Husbandry University, Nyingchi 860000, China

**Keywords:** 离子色谱, 中草药, 复杂组分, 成分分析, ion chromatography (IC), Chinese herbal medicine, complex components, component analysis

## Abstract

离子色谱是用于分离分析不同基质样品中离子性物质的一种新型高效液相色谱技术。自1975年发展至今,已被广泛应用于环境、能源、食品、医药等多个领域,具有操作简单、分析快速、灵敏度和选择性高,且能同时分离测定多种组分等优点。近年来,随着离子色谱技术自身迭代发展,可测定分析的样品种类已包括离子、糖类、氨基酸、有机酸(碱)等,同时离子色谱法也越来越成为针对中草药复杂组分中单个有效成分分析与鉴定的重要手段。本文介绍了离子色谱技术的不同类型、原理及研究进展,整理了近几十年离子色谱在中草药糖苷类、氨基酸、蛋白质、无机盐以及有机酸、生物碱类和黄酮类等复杂成分中的应用情况;检索文献发现,离子交换色谱、电导检测法为离子色谱中最常用的技术类型和检测方式,且目前离子色谱在生物碱类成分分析中的应用展现出较传统分析方法更好的优势,但在无机阴离子的形态分析和黄酮类、苯丙素类、甾体类等主要活性物质中的直接应用研究报道较少。最后,综述了离子色谱(联用)新技术及其在中草药中的最新进展,并对该色谱方法未来在复杂组分分离分析方面的应用进行了探讨和展望,为离子色谱技术分析中草药复杂化学成分的进一步发展提供理论参考。

## Abstract (en)

Ion chromatography (IC) is a novel high performance liquid chromatographic technique that is suitable for the separation and analysis of ionic substances in different matrix samples. Since 1975, it has been widely used in many fields, such as the environment, energy, food, and medicine. IC compensates for the separation limitations of traditional gas chromatography and high performance liquid chromatography and can realize the qualitative analysis and quantitative detection of strongly polar components. This chromatographic technique features not only simple operations but also rapid analysis. The sensors used in IC are characterized by high sensitivity and selectivity, and the technique can simultaneously separate and determine multiple components. Several advances in IC instrumentation and chromatographic theories have been developed in recent years. IC can analyze various types of samples, including ions, sugars, amino acids, and organic acids (bases). Chinese herbal medicines are typically characterized by highly complex chemical compositions and may contain carbohydrates, proteins, alkaloids, and other active components. They also contain toxic residues such as sulfur dioxide, which may be produced during the processing of medicinal materials. Therefore, the analysis and elucidation of the precise chemical constituents of Chinese herbal medicines present key problems that must be resolved in modern Chinese herbal medicine research. In this context, IC has become an important method for analyzing and identifying the complex components of Chinese herbal medicines because this method is suitable for detecting a single active ingredients among complex components.

This paper introduces the different types and principles of IC as well as research progress in this technique. As the applications of IC-based methods in pharmaceutical science, cell biology, and microbiology increase, further development is necessary to expand the applications of this technique. The development of innovative techniques has enabled IC technologies to achieve higher analytical sensitivity, better selectivity, and wider application. The components of Chinese herbal medicines can be divided into endogenous and exogenous components according to their source: endogenous components include glycosides, amino acids, and organic acids, while exogenous components include toxic residues such as sulfur dioxide. Next, the applications of IC to the complex components of Chinese herbal medicines in recent decades are summarized. The most commonly used IC technologies and methods include ion exchange chromatography and conductivity detection. The advantages of IC for the analysis of alkaloids have been demonstrated. This method exhibits better characteristics than traditional analytical methods. However, the applications of IC for the speciation analysis of inorganic anions are limited. Moreover, few reports on the direct application of the technique for the determination of the main active substances in Chinese herbal medicines, including flavonoids, phenylpropanoids, and steroids, have been reported.

Finally, this paper reviews new IC technologies and their application progress in Chinese herbal medicine, focusing on their prospects for the effective separation and analysis of complex components. In particular, we discuss the available sample (on-line) pretreatment technologies and explore possible technologies for the selective and efficient enrichment and separation of different components. Next, we assess innovative research on solid-phase materials that can improve the separation effect and analytical sensitivity of IC. We also describe the features of multidimensional chromatography, which combines the advantages of various chromatographic techniques. This review provides a theoretical reference for the further development of IC technology for the analysis of the complex chemical components of Chinese herbal medicines.

离子色谱(ion chromatography, IC)又称高效离子色谱(HPIC)、现代离子色谱,于1975年由Dow化学公司化学家Small等^[1]^首次提出,是基于离子性化合物与固定相表面离子性功能基团之间的电荷相互作用,实现离子性物质分离和分析的高效液相色谱(HPLC)技术。IC发展初期,主要用于测定样品中的无机阴离子^[2]^,后来随着色谱仪器更新迭代及色谱理论深入发展,可测定分析的样品种类已增加至糖类、氨基酸、有机酸(碱)、金属离子等^[3⇓⇓-6]^,成为多种常见离子的标准检验方法^[7]^。

中草药化学成分十分复杂^[8]^,包含自有的糖类、蛋白质类、生物碱类等有效成分及在药材处理过程中可能产生的二氧化硫等有毒残留物质,因此分析和阐明其精准化学成分一直是现代中草药研究中亟待解决的关键问题。IC弥补了传统气相色谱(GC)^[9]^和HPLC^[10]^的分离弱势,可实现对强极性成分的定性分离和定量检测,具有高灵敏度、高选择性、操作快速简单、能同时分离测定多种组分等优点^[11]^。2010年,IC首次被《中国药典》收载入附录中,成为中草药成分分析及检验领域十分重要的分析手段。基于此,本文综述了近年来IC及其新技术在中草药成分分析领域的应用,为扩展其在中药、民族药复杂组分分析与鉴定方面的应用提供理论参考。

## 1 IC的理论概述及研究进展

### 1.1 IC理论概述

IC分析仪器的组成包括流动相输送系统、进样系统、分离系统、检测系统和数据采集分析系统等(见图1),依据分离机制不同,IC主要分为离子交换色谱(ion exchange chromatography, IEC)、离子对色谱(ion pair chromatography, IPC)和离子排斥色谱(ion exclusion chromatography, ICE)^[12,13]^。其中IEC是IC中应用最广泛的分离方式,它利用离子交换的原理,通过不同离子与固定相间的库仑力差异达到分离定性的目的^[11]^,适用于中草药中亲水性阴阳离子的分析^[14]^; IPC的分离原理是基于待测组分在分离柱上的吸附分配作用^[15]^,通常采用普通HPLC的分离体系,适用于分析具有表面活性的阴阳离子及金属络合物等强极性成分^[16,17]^; ICE主要依据Donnan排斥效应、空间排斥和吸附作用,利用待测组分与固定相之间的非离子性相互作用机制进行分离^[18,19]^,适用于有机酸、无机弱酸及醛、醇、氨基酸等成分的分离分析^[20,21]^。

**图 1:**
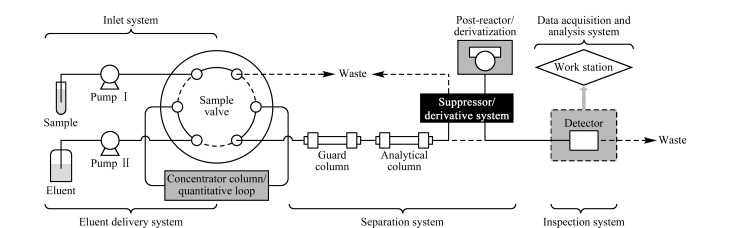
IC技术流程示意简图

IC检测器分为两大类,即电化学检测器和光学检测器。使用电化学检测器的IC检测技术包括最通用的抑制电导检测法、直接电导检测法、电化学安培法(直流、脉冲、积分脉冲安培法)等;而紫外-可见吸收光度法、荧光法等是应用光学检测器的IC检测方式。此外,IC还可与质谱(MS)、电感耦合等离子体(ICP)等联用(IC-MS、IC-ICP-MS^[22,23]^)以适用更加广泛的分析领域。

电导检测法作为IC中最常用的检测方法,分为抑制电导和直接电导,是通过测量缓冲溶液中分析物离子与共存离子的摩尔电导率来产生响应信号^[24]^,常见的无机、有机阴阳离子^[25,26]^和生物化学物质(如氨基酸)^[27]^等都可用此种方法。电化学安培法是在工作电极上对分析物进行氧化还原反应,可选择性检测S ${O_{3}}^{2-}$ 、N ${O_{2}}^{-}$ 、CN^-^、I^-^、S^2-^等无机阴离子以及有机胺、醛、醇、酚等有机离子。紫外-可见吸收光度法具有高灵敏度和高选择性,可测定在紫外或可见光区域内有吸收的N ${O_{2}}^{-}$ 、I ${O_{3}}^{-}$ 和Br ${O_{3}}^{-[28]}$ ;而间接紫外检测常用于自身不具有紫外吸收离子的分析,其流动相一般由具有强紫外吸收的苯多羧酸酯类化合物(benzenepolycarboxylates)组成^[29]^,检测信号为负值。荧光检测是另一种基于光发射的技术,除可直接应用于具有天然荧光特性的分析物或经过适当柱前衍生化的分析物外,亦会以柱后反应模式进行分析物测定(柱后衍生光度法),可选择性检测氨基酸及Hg^2+^、Pb^2+^、Cr^6+^等重金属和稀有金属离子^[30⇓-32]^。IC与元素(结构)选择性检测器(如等离子体发射光谱(Plasma-OES)、原子吸收光谱(AAS)等)联用综合了分离性好、选择性高和灵敏度高的优势,为某些特定元素的形态分析提供了合适方法^[33]^。

### 1.2 IC研究进展

IC作为现代实验室中重要的色谱分析技术,近年来在研究和应用领域得到了广泛关注,并取得显著进展。迄今为止,IC除在环境、食品、材料科学等领域的应用外,在药物科学、细胞生物学、微生物学等领域的应用也越来越多,具有进一步发展和应用的空间(见图2)。此外,IC新技术的发展同时为复杂组分研究奠定了硬件基础,主要体现在以下方面:①IC-MS技术。通过结合IC的分离能力和MS的检测灵敏度,IC-MS能够实现对复杂离子混合物的高灵敏度和高选择性检测。②IC的微流控技术。通过采用微流控芯片的样品制备方法,将分析物经处理、分离、检测等基本操作单元集成或基本集成到芯片中,实现了高效的分离、快速的分析和低样品消耗。③新型IC固定相材料。通过不断开发和改进具有较大表面积、高化学活性和更好稳定性的新型固定相材料,如金属有机骨架(metal-organic frameworks, MOFs)、离子液体和纳米材料等,可以提高IC的分离效果和分析灵敏度。④IC的在线前处理技术。能够实现对分析物中的离子化合物进行选择性富集和分离,提高IC的灵敏度和选择性。

**图 2:**
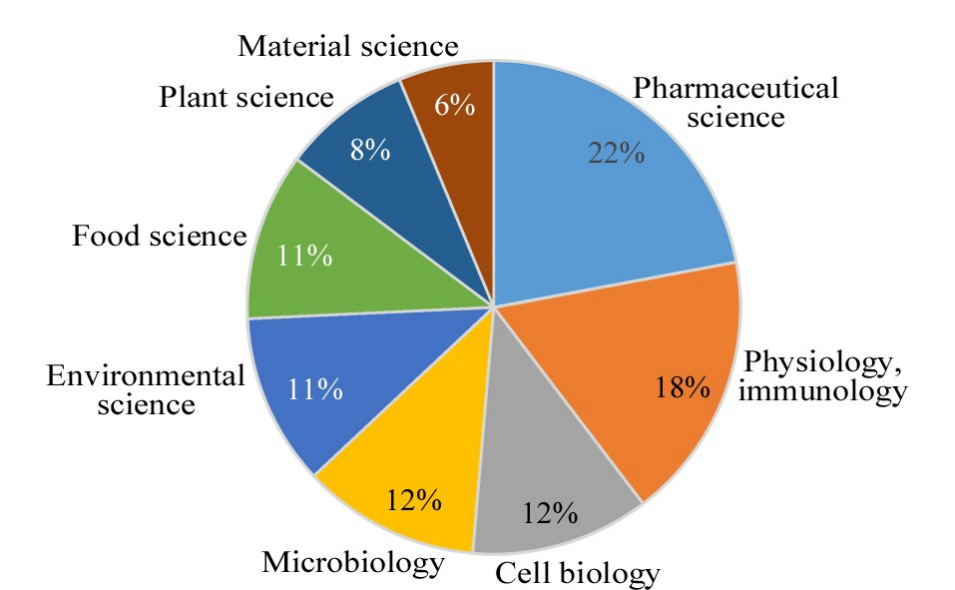
1975年9月至2023年11月IC应用的不同研究领域及其相关论文发表比例(基于Web of Science检索)

IC-MS联用技术的发展、微流控技术的应用、新型固定相材料的研究和在线前处理技术的创新等方面的进展,使IC具备了更高的分析灵敏度、更好的选择性和更广阔的应用领域。随着技术的不断发展和创新,IC将扮演更重要的角色,为科学研究和实际应用提供更多可能。

## 2 IC在中草药中的实际应用

中草药的化学成分极为复杂,以来源不同可分为内源性成分和外源性成分。在内源性成分中有些是一般药用植物普遍存在的^[34]^,如糖苷类、氨基酸、蛋白质、无机盐和微量元素;另一部分则是同科或同属植物某些器官中特殊共存的有机化合物^[35]^,如某种有机酸类、生物碱类、黄酮类等,并且大多具有显著的药理活性。此外,还包括中药材处理过程中可能产生的二氧化硫等有毒残留物质。本部分总结了IC发展至今在中草药内源性成分和外源性成分分析中的应用。

### 2.1 中草药内源性成分的分析

#### 2.1.1 糖苷类物质的分析

中草药中富含的糖苷类化合物是中草药重要的生物活性物质之一。先前对于糖类物质的分析方法主要有GC、HPLC、亲和色谱与HPLC联用、毛细管电泳法(CE)等,而后鉴于糖类成分在强碱溶液中可呈离子化状态且拥有电化学活泼性的原理^[36]^, IC便发展成为其重要分析方法之一。例如,Sekiguchi等^[37]^使用Dionex CarboPac PA1、CarboPac PA1 guard色谱分析柱,以75 mmol/L NaOH和75 mmol/L NaOH-500 mmol/L乙酸钠为流动相梯度淋洗,流速为1 mL/min,电化学脉冲安培法检测分析了药用植物拟南芥中的磷酸糖。该法同时使用Titansphere TiO柱富集纯化磷酸糖,提高了对磷酸糖的分辨率,减少了植物提取物中未知化合物的干扰,为测定复杂成分中的磷酸糖提供了简易方法。乐胜锋等^[38]^采用Dionex CarboPac PA10色谱柱分离,14~20 mmol/L NaOH以1 mL/min流速梯度洗脱,电化学积分脉冲安培法测定了芦荟多糖中岩藻糖、鼠李糖等7种单糖,使常见多糖与单糖的定性定量分析方法得到优化。许玮仪等^[39]^使用Dionex Carbopac PA210-Fast-4μm分析柱,以积分脉冲安培法测定了肉苁蓉多糖中单糖的组成和游离单糖的含量,该法较1-苯基-3-甲基-5-吡唑啉酮柱前衍生HPLC方法的前处理过程简单,在肉苁蓉果糖组成测定时更有优势。Wang等^[40]^在莲花多糖的提取纯化研究中提到离子交换色谱也是目前纯化和分离多糖物质的最常用技术。

另外,对于糖的衍生物-苷类物质的分析,IC也展现出独特优势。例如,刘鹏等^[41]^建立了反相离子对色谱,采用Agilent Eclipse XDB-C18色谱柱,以乙腈-0.15%磷酸二氢钠溶液为流动相,在波长203 nm处测定了苍耳子中2种苍术苷毒性成分。该方法具有快速可靠、耐用性好、专属性强等特点,使羟基苍术苷、苍术苷在20 min内即得到了良好分离。

#### 2.1.2 氨基酸的分析

氨基酸是生物大分子蛋白质和酶的基本组成单元,主要以游离态、结合态2种形式存在于自然界中。中草药中富含大量人体所需的氨基酸,它是为人类提供营养及发挥药效的关键组分,因此,氨基酸的分析也是中草药复杂成分分析中的重要部分^[42]^。目前氨基酸的分析方法主要有化学分析法^[43]^、光谱分析法^[44]^、色谱分析法和CE^[45]^等,而色谱分析法中的IEC与IPC又是分析氨基酸组分通常使用的2种高效IC。例如栾兰等^[46]^选择日立2622 SC型阳离子交换树脂色谱柱,使用5种缓冲溶液以0.28 mL/min的流速梯度洗脱,以茚三酮为衍生显色剂,显色剂流速为0.32 mL/min;检测波长设定为570、440 nm(针对脯氨酸和羟脯氨酸),测定了金银花、连翘和黄芩3种药材中游离及水解后的氨基酸含量。该法(柱后衍生-阳离子交换色谱法)利用氨基酸自动分析仪建立其色谱分离方法,得到各药材中游离及总氨基酸的含量,检测结果准确。彭涛等^[47]^将LCA K 06/Na阳离子分离色谱柱作为分析柱,以柠檬酸缓冲溶液(CPBS, pH 3.45)、柠檬酸硼酸缓冲溶液(CP-BBS, pH 10.85)和0.02%乙二胺四乙酸碱性溶液为流动相梯度洗脱,建立了氨基酸分析仪-柱后衍生离子交换色谱测定红芪中天冬氨酸、丝氨酸、组氨酸等17种氨基酸的方法。朱岩等^[48]^研发了一种IC-抑制电导检测白氨酸、牛磺酸、苯丙氨酸和苯酚等氨基酸类成分的方法,以0.75 mmol/L KCl+NaOH (pH 11.5)为淋洗液在2 mL/min的流速下等度洗脱。而后刘春梅等^[49]^又使用IC-直接电导检测法测定了混合标样中的苏氨酸含量。该方法检测样品可不经衍生化处理,直接分析,具有操作简便、检测迅速及结果可靠等特点。

此外,Clarke等^[50]^提出的使用高效阴离子交换色谱-积分脉冲安培法测定氨基酸的技术也成为氨基酸分析的常用方法,该方法具有灵敏性高、稳定性好等优点,对于大多数氨基酸的检出限通常能够达到pmol或fmol级^[51]^。如钟添华等^[52]^用Dionex AminoPac PA10型分析柱,以H_2_O、250 mmol/L NaOH和1 mol/L NaAc为流动相进行梯度洗脱,流速为0.25 mL/min,电化学积分脉冲安培法分析测定了药材金线莲中的17种氨基酸。

除IEC外,IPC在中草药氨基酸的分离分析方面同样得到了广泛应用^[53]^。例如,Moldoveanu等^[54]^采用离子对色谱法分离样品中的不同种氨基酸,然后再用质谱检测器分析测定了18种药用植物样品中的游离氨基酸含量。

#### 2.1.3 蛋白质和酶的分析

蛋白质是自然界中的一类大分子化合物,广泛存在于生物体的各种组织和细胞中。研究已发现,中草药中的某些蛋白质(如天花粉蛋白、槲寄生凝集素、豆豉纤溶酶等)是其发挥抗病毒、抗肿瘤、抗氧化及免疫调节等药理活性的重要有效成分之一^[55]^。酶作为一类催化生物化学反应的特殊蛋白质,也常存在于中草药中。目前在蛋白质的分析中,通常会采用电泳、光谱、色谱及质谱等技术,其中色谱技术是分离纯化植物中蛋白质类成分的主要分析方法^[56]^,而作为色谱技术之一的离子交换色谱也是最经典的一种蛋白质纯化分析手段。例如,Haq等^[57]^利用DEAE Sephadex A50离子交换色谱柱分离纯化了茴香叶黑种草中的全部蛋白质类组分。该法以0.01~2.00 mol/L NaCl和0.05 mol/L磷酸盐缓冲液(PBS, pH 6.4)为淋洗液线性梯度洗脱,于检测波长280 nm处测定分析得四组馏分,而后通过十二烷基硫酸钠-聚丙烯酰胺凝胶电泳对其进行分析,结果显示出许多(94±10) kDa的蛋白质条带。Jiang等^[58]^亦采用DEAE-Sepharose离子交换色谱,再联合Sepharose 4B亲和色谱、Sephacryl S-100凝胶过滤色谱等方法从中草药见血青的根茎中分离纯化甘露糖结合凝集素。经鉴定,纯化得到的*L. nervosa*凝集素(LNL)为一个单体蛋白(13 kDa)。Mulla等^[59]^通过超速离心过滤-离子交换色谱法提取疣柄魔芋块茎中的酪氨酸酶。该方法是依据Balkrishnan的酶提取法先将疣柄魔芋块茎切成小块,在4 ℃下用搅拌器将其匀浆到50 mmol/L PBS(pH 6.0)中,再经超速离心、过滤得到滤液部分后,采用DEAE-cellulose色谱柱,以0~300 mmol/L NaCl为流动相线性梯度洗脱,最终得到了高度纯净的酪氨酸酶(纯化倍数为12.65,比活力为60.25 U/mg)。

#### 2.1.4 无机盐的分析

中草药中含有丰富的无机盐,它们一般是由金属阳离子与无机阴离子组成,其中金属阳离子的元素类型可分为常量元素(K、Ca、Mg等)和微量元素(Zn、Fe、Mu等),这些金属元素对中药材中多种生物分子的活性发挥起关键调控作用,可协同促进某些有效成分发挥药理作用^[60⇓-62]^。近年来,中药材中无机阴离子与其疗效的关系也日益受到关注,对其阴离子的分析测定可作为评价中药质量的重要参考。这些阴阳离子等无机元素与中草药的质量优劣及药性、药效都有着密切关系,因此研究其高效的分离分析方法具有重要意义^[63]^。无机阴阳离子的分析方法主要有化学分析法、光度分析法、色谱分析法等,IC相较于以往传统的化学分析方法、仪器分析方法具有操作简便、分析快速和灵敏度高的优势^[64]^,已逐渐成为测定中药材中常见阴阳离子的首选方法。

首先,在金属阳离子的测定方面,田甜等^[65]^使用IonPac CS12A分析柱、CSRS 4mm抑制器,以20 mmol/L甲烷磺酸(MSA)水溶液为淋洗液,检测了石膏-知母组合物中Na^+^、K^+^、Mg^2+^、Ca^2+^,并建立了相应的指纹图谱。王宗花等^[66]^以ICS-C25阳离子交换柱为分析柱,在2.5 mmol/L的均苯四甲酸淋洗液下等度洗脱,流速选择0.6 mL/min,抑制电导检测快速分析了川芎和酸枣仁提取液中的Na^+^、N ${H_{4}}^{+}$ 、K^+^、Mg^2+^和Ca^2+^等5种常见金属阳离子的含量。该法中各离子的最低检出限为0.001~0.013 mg/L,线性范围可达3个数量级。Yelampalli等^[67]^采用Dionex^®^ IonPac^®^ CS16为色谱柱,6.7% MSA水溶液为流动相,流速为1.2 mL/min,对口服制剂中的无机盐(Na_2_SO_4_、K_2_SO_4_、MgSO_4_)进行定量分析。结果显示,钠、钾和镁硫酸盐的保留时间分别为7.8、12.8和16.2 min,并通过ICH(The International Council for Harmonisation of Technical Requirements for Pharmaceuticals for Human Use,人用药品技术要求国际协调理事会)指南进行验证,得到良好的线性关系和准确度,钠、钾和镁硫酸盐的线性范围分别为80.0~240.0、20.0~60.0和4.5~13.5 mg/L(ppm)。郭新苗等^[68]^亦使用IonPac CS12A分析柱,以20 mmol/L MSA为淋洗液对金银花提取物进行等度洗脱,准确测定了金银花中K^+^、Na^+^、Mg^2+^和Ca^2+^等4种金属阳离子的含量,检出限分别为0.025、0.020、0.050和0.015 mg/L。此方法快速、灵敏度高、结果可靠,为中药金银花中阳离子的含量测定提供了一种有效方法。其次,在无机阴离子测定中,展建丽等^[69]^则采用Dionex IonPac AS22型色谱柱,以超纯水-NaHCO_3_ (1.5 mmol/L)-Na_2_CO_3_ (4.5 mmol/L)溶液为流动相等度洗脱,抑制电导检测法测定了黄花草木犀中Cl^-^、N ${O_{3}}^{-}$ 和S ${O_{4}}^{2-}$ 等3种无机阴离子的含量,结果显示各离子浓度与峰面积具有良好的线性关系,适宜测定黄花草木犀中阴离子的含量。张元等^[70]^利用IC-抑制电导检测法测定了丹参注射剂中F^-^等6种阴离子的含量。该方法以IonPac AS11-HC阴离子色谱柱为分析柱,KOH溶液为淋洗液梯度洗脱(运行0~13 min,浓度变化为12~20 mmol/L),为丹参注射剂的质量控制研究提供了一种简便可靠的方法。李冰茹等^[71]^将三七药材经水浴提取,正己烷净化,使用IonPac AS15型色谱柱为分析柱,以30 mmol/L KOH溶液为洗脱剂等度淋洗,通过抑制电导检测器分析测定了三七中Cl^-^的含量,与高温干法灰化法相比精密度更优。除抑制电导检测方法外,王宗花等^[72]^还采用非抑制型阴离子交换色谱法,以Shim-pack IC-A1为色谱柱,0.8 mmol/L邻苯二甲酸氢钾溶液为淋洗液,快速分离分析了川芎提取液中的H_2_P ${O_{4}}^{-}$ 等3种无机阴离子。该方法使3种离子得到了较好分离,Cl^-^、N ${O_{3}}^{-}$ 和H_2_P ${O_{4}}^{-}$ 的检出限分别为0.05、0.18、0.71 mg/L。

此外,IC还可用于金属阳离子中某些微量元素(如砷、铬、锑等)的形态分析^[73]^以及部分常见无机阴离子的初级形态分析。例如,侯逸众等^[74]^建立了IC-双阳极电化学氢化物发生-原子荧光光谱法测定当归中锑元素的形态,该法实用性较强,方法准确可靠。Yang等^[75]^采用IC测定了阿胶中的痕量六价铬(Cr(Ⅵ));他们首先基于聚合物的反相色谱预处理柱保留样品中复杂的有机化合物基质,之后通过阀切换技术将带有目标离子的样品溶液输送到IonPac AS19分析柱中,在波长545 nm处进行检测。王小平等^[76]^通过建立IC-ICP-MS的方法对松茸等植物中6种砷的形态分布开展了分析,得知其生物体内总砷含量最高,而无机砷(亚砷酸+砷酸)占总砷比例最低(3.7%~6.8%),砷甜菜碱占比最高(75.8%~87.3%);该方法可在较短时间内实现各类砷化合物的较好分离,且峰形较好,检出限和定量限均分别不超过0.005 mg/kg和0.02 mg/kg。徐万帮等^[77]^也通过IC-ICP-MS联用方法对沉香化气丸中的6种砷和铬开展形态分析。近年来,对金属元素形态分析的相关研究较为丰富,但针对中草药中非金属成分(无机阴离子)的分析研究相对缺失,时嵩年等^[78]^首次采用IC对中草药中几种常见无机阴离子的形态进行了分析研究,但该研究也只包括了可溶态、悬浮态(中草药水煎液经0.45 μm滤膜过滤分离所得),是初级的形态分析。

#### 2.1.5 有机酸的分析

有机酸指含有羧基(-COOH)的化合物,是中草药中一类重要的组成成分,具有抗炎、抗氧化、抑菌及抗惊厥等多种药理作用^[79⇓-81]^,常见的有机酸类物质有草酸、柠檬酸、苹果酸、绿原酸等,常用的分析方法包括RP-HPLC、GC、薄层色谱(TLC)和IC等^[82]^。在IC的3种类型中,有机酸的分离分析主要应用的是IEC与ICE。有机酸的IEC是利用其在水溶液中的可解离性差异而达到分离目的,通常来说,越易离解的成分在离子交换色谱柱上的保留值越大。例如,Li等^[83]^开发了一种阴离子交换色谱柱同时分离测定黄连中7种有机酸的方法,其采用IonPac AS11-HC色谱柱,以KOH溶液为流动相梯度洗脱,采用抑制电导法检测了奎宁酸、乙酸、甲酸、酒石酸、苹果酸、琥珀酸和草酸,结果表明有机酸可作为黄连药材品质辅助鉴定的有效成分。夏雪琴等^[84]^采用Dionex IonPac AS19分离柱,以4~54 mmol/L KOH为流动相梯度洗脱,同时测定了乌梅及其配方颗粒中的草酸、柠檬酸等4种有机酸类物质的含量。该方法中有机酸成分的分离度良好,可应用于快速检测乌梅等富含有机酸类物质的药材。华丰等^[85]^用Transgenomic ICSep Ion 300色谱柱分离,8 mmol/L H_2_SO_4_溶液等度淋洗,流速为0.5 mL/min,抑制电导检测法测定了灵芝提取物中的4种有机酸,结果显示在1~50 μg/mL范围内具有较好的线性关系,加标回收率为98.2%~100.4%, 相对标准偏差(RSD)均小于2%。

在ICE中,有机酸类的分离主要受溶液中Donnan排斥效应的支配,其在色谱柱上的保留情况恰与IEC相反。例如,刘瑞等^[86]^使用Transgenomic Sep-ION-300分离柱,以5 mmol/L H_2_SO_4_-丙酮(95∶5, v/v)为淋洗液等度洗脱,电导检测法分析了生脉注射液中的柠檬酸、苹果酸和琥珀酸。该法所需样品前处理简单,具有可靠性高、重现性好的特点。林晓婕等^[87]^采用IC-Pak Ion Exclusion色谱柱,以H_2_SO_4_-乙腈(98∶2, v/v)混合液为流动相,并设定H_2_SO_4_浓度的线性梯度程序(0~40 min, 0.01~0.02 mol/L; 40~50 min, 0.01 mol/L),于210 nm处快速分离检测了黄酒中的草酸、酒石酸、抗坏血酸等13种有机酸,且在0.001~1.000 g/L范围内具有良好的线性关系。

#### 2.1.6 生物碱类物质的分析

生物碱是广泛存在于茄科、毛茛科和小檗科等多种植物类中药材中的一类含氮天然有机化合物,多具有显著而特殊的生理活性^[88]^,如小檗碱、苦参碱等具有抗肿瘤作用^[89]^;阿托品、东莨菪碱等具有解痉作用^[90]^;吗啡、延胡索乙素等有镇痛作用^[91]^。目前,针对中草药中生物碱类成分的检测方法主要有重量法、薄层扫描法、CE、HPLC和IC等^[92,93]^,而IC在生物碱分析测定的众多方法中展现出更好的优势。例如,曾文芳等^[94]^采用碳纳米管修饰电极离子交换色谱法测定从中药麻黄中提取的盐酸麻黄碱,该方法使用Dionex AG9-HC分析柱,以8 mmol/L Na_2_CO_3_-甲醇(9∶1, v/v)混合液为淋洗液,电化学直流安培法检测;结果显示,当使用未修饰电极测定盐酸麻黄碱时检出限为2.4 μg/L,而修饰后检出限降至0.2 μg/L,方法灵敏度提高了10倍以上,为盐酸麻黄碱含量测定的新方法研究提供了参考。张晨光等^[95]^用WY-Cation-1阳离子分析柱,以2.0 mmol/L MSA溶液为流动相等度淋洗,直接电导检测法测定糖蜜提取物中的甜菜碱。结果表明,甜菜碱在0.5~50 mg/L范围内具有良好的线性关系,检出限为0.15 mg/L。除运用离子交换色谱法外,离子对色谱法也常用于生物碱类化合物的检测,鄢丹等^[96]^采用反相离子对色谱法同时测定左金丸中盐酸小檗碱、吴茱萸碱、硫酸黄连碱等7种生物碱的含量。该方法以Diamonsil C18为分析柱,1.5 mmol/L十二烷基硫酸钠溶液和乙腈为洗脱剂梯度淋洗,于紫外检测波长265 nm处分析测定,结果显示各生物碱进样浓度与色谱峰面积具有良好的线性关系,为含吴茱萸、黄连的复方制剂的质控提供参考。陈晨等^[97]^亦运用此方法同时测定了复方苦参注射液中苦参碱、氧化苦参碱、槐果碱和氧化槐果碱4种生物碱的含量。

此外,IC与其他技术联用使得对中草药复杂成分的分析效率及灵敏度进一步提高,如Feng等^[98]^建立了一个IEC、亲水相互作用色谱(HILIC)和反相色谱(RPC)相结合的三维液相色谱(3D-LC)系统测定药用植物白钩藤中复杂的生物碱类成分;在一维IEC系统中,该法采用PhenoSphere^TM^SCX色谱柱,以20 mmol/L乙酸铵-0.05%甲酸(FA)水溶液和甲醇为流动相等度洗脱,紫外检测器设置在254 nm和203 nm处,分别检测生物碱和三萜酸,之后再通过二维HILIC、三维RPC对各组分进行分离分析,结果共分离到308种成分,鉴定或初步表征了128种成分(其中生物碱85种、三萜酸类(TAs)29种、其他14种)。这种3D-LC系统具有较高的精密度(日内RSD < 5%),优于传统的一维LC-MS系统。

#### 2.1.7 黄酮类物质的分析

黄酮类化合物来源广泛,是自然界中以C6-C3-C6为基本碳骨架的多类型有机化合物,主要分布于唇形科、菊科、玄参科等植物中,大多以糖苷形式存在,部分以游离形式存在^[99]^。同时,该类物质也是中草药中一类非常重要的有效成分,具有多方面的药理作用,如花青素、黄芩苷、槲皮素等具有抗炎、抗氧化作用,临床上可用于治疗心血管疾病^[100]^;水飞蓟素等具有肝保护作用,20世纪70年代已被世界卫生组织列为治疗肝损伤疾病的官方药物^[101]^;川陈皮素、杜鹃素等还具有止咳化痰的作用。目前分析黄酮类物质的方法主要有薄层扫描法、分光光度法、HPLC和HPLC-MS联用的方法等^[102]^。通过大量的文献检索发现,IC作为一种新型技术,有关其在黄酮类化合物中分析应用的报道很少,李清潭等^[103]^也只是建立了一种以D941树脂为分离柱填料的连续离子交换色谱分离甘草提取液中甘草黄酮和甘草酸的最佳工艺,该方法初步探究了黄酮类物质分离的色谱柱类型,为后续IC在黄酮类化合物分析应用研究提供了参考。

### 2.2 中草药外源性成分的分析

中草药在加工处理过程中常会采用硫黄熏蒸技术,该方法在我国已实际应用了很长时间,不仅能够有效防止中药材腐烂、霉变和虫蛀,而且可以将药材漂白增艳,改善品质^[104]^。但是依赖这种传统方法处理中草药,极易使熏蒸过程中产生的非自有成分(亚硫酸盐物质)残留在药材中,这些亚硫酸盐物质(也称二氧化硫残留)一方面会造成中药材中的某些有效成分(如苷类、生物碱等^[105,106]^)发生量变或质变,另一方面过量摄入也会对人体造成一定的危害,如头痛、恶心,甚至神经系统损伤等^[107,108]^。因此,探究建立精准高效检测中草药中二氧化硫残留的新方法,对中药质量、安全性评价及人类用药安全具有重要意义。目前检测中草药中二氧化硫残留量的常用方法主要有滴定法、分光光度法、GC和IC等^[109]^,其中IC以高灵敏度和高准确性的特点在众多测定方法中凸显独特优势。例如,皮文霞等^[110]^比较了滴定法与IC测定白术、山药、苦杏仁、金银花、熟党参等5种中草药中二氧化硫残留量的结果和适用性,选用IonPac@AS11-HC为分析柱,以20 mmol/L KOH溶液为淋洗液等度洗脱,抑制电导检测法测定样品中的二氧化硫残留量。结果显示滴定法的干扰因素较多,误差较大;而IC具有灵敏度高、专属性强等优点,可用于中药材二氧化硫残留量的准确测定,但在实际工作中还应根据处理条件及中草药品种差异选择不同方法检测。饶毅等^[111]^使用IonPac AS18型离子交换柱,NaOH水溶液为洗脱剂,流速为0.25 mL/min,测得大黄中二氧化硫残留量为(189.92±7.14) μg/g,建立了适用于IC标准曲线法测定中草药中二氧化硫残留量的不确定度分析方法。谭聪等^[112]^采用Dionex Ionpac AS11A型阴离子交换色谱柱测定中药苦杏仁和桃仁中的二氧化硫残留量,以12 mmol/L KOH溶液为洗脱剂,流速为1.2 mL/min等度淋洗,测得残留量为24.78~88.57 μg/g。本法检测快速简单,能够避免假阳性干扰,可用于含有苦杏仁苷类成分的中药材中二氧化硫残留量的测定。此外,李继等^[113]^还选择3.2 mmol/L Na_2_CO_3_和1.0 mmol/L NaHCO_3_溶液作为淋洗液,以0.7 mL/min的流速等度淋洗,用A-SUPP5-150型IC柱测定中药天麻中的二氧化硫残留,测得其平均含量为241.3 mg/kg,平均回收率为98.0%,并通过单因素实验,探究了天麻经不同方法蒸煮所得的二氧化硫蒸出量差异,结果显示二氧化硫的蒸出量与HCl溶液浓度和蒸煮时间有关联。李耀磊等^[114]^使用IonPac AS-11-HC阴离子交换柱为分析柱,以20 mmol/L KOH溶液为淋洗液,抑制电导检测法检测分析了菊苣根中的二氧化硫残留量(7.8~46.3 mg/kg),说明该中药材中二氧化硫残留风险整体较低,符合国家标准。

综上,离子色谱在中草药成分分析中的应用见表1。

**表 1:** IC在中草药成分分析中的应用

No.	Sources	Analytes				Columns			Eluents^*^			Detection			IC type			Ref.		
1	Arabidopsis thaliana	glucosides (sugar phosphates)				Dionex CarboPac PA1, CarboPac PA1 guard			E1: 75 mmol/L NaOH, E2: 75 mmol/L NaOH, 500 mmol/L NaAc			ECAM (PAD)			IEC			[37]		
2	aloes	glucosides (fucose, rhamnose, arabinose, galactose, glucose, mannose, xylose)				Dionex CarboPac PA10			14-20 mmol/L NaOH			ECAM (integrated PAD)			IEC			[38]		
3	Cistanches herba	glucosides (fucose, rhamnose, arabinose, galactose, glucose, mannose, xylose, fructose, ribose)				Dionex Carbopac PA210-Fast-4μm			20, 200 mmol/L NaOH			ECAM (integrated PAD)			IEC			[39]		
4	Xanthii Fructus	glucosides (carboxyatractyloside, atractylodis)				Agilent Eclipse XDB-C18			ACN-0.15% NaH_2_PO_4_ (containing 0.12% TBAH, adjusted to pH 3.5 with H_3_PO_4_)			UV-Vis (203 nm)			IPC			[41]		
5	Lonicerae Japonicae flos, Forsythiae fructus, Sectellarlae radix	amino acids				Hitachi 2622 SC			5 buffer solutions			UV-Vis (570, 440 nm)			IEC			[46]		
6	Hedysari Radix	amino acids (aspartic acid, serine, histidine, etc.)				LCA K 06/Na			A: pH 3.45 CPBS, B: pH 10.85 CP-BBS, C: 0.02% EDTA alkaline solution			UV-Vis (570, 440 nm)			IEC			[47]		
7	mixed standard solution	amino acids (leucine, taurine, phenylalanine)				AG4A-SC(DX-100T IC)			0.75 mmol/L KCl+ NaOH (pH 11.5)			suppressor CD			IEC			[48]		
8	mixed standard solution	amino acid (threonine)				Metrosep C2-150			4 mmol/L tartaric acid, 1 mmol/L DPA			direct CD			IEC			[49]		
9	mixed standard solution	amino acids				AminoPac PA10			60-200 mmol/L NaOH, 400 mmol/L NaAc			ECAM (PAD)			IEC			[50]		
10	Anoectochilus roxburghii	amino acids (arginine, alanine, glycine, valine etc.)				Dionex AminoPac PA10			A: water, B: 250 mmol/L NaOH, C: 1 mol/L NaAc			ECAM (PAD)			IEC			[52]		
11	18 medicinal plant samples	amino acids (19 species)				Dionex Acclaim^TM^ RSLC Polar Advantage Ⅱ			A: water containing 1% ACN-0.5% HFBA- 0.02% TFA, B: ACN containing 0.1% TFA			IC-MS			IPC			[54]		
12	Nigella sativa	proteins				DEAE-Sephadex A50			50 mmol/L PBS (pH 6.4), 0.01-2 mol/L NaCl			UV-Vis (280 nm)			IEC			[57]		
13	Liparis nervosa	protein (mannose-binding lectin)				DEAE-Sepharose			50 mmol/L PBS (pH 8.5), 0-1 mol/L NaCl			UV-Vis (280 nm)			IEC			[58]		
14	Amorphophallus paeoniifolius	protein (tyrosinase)				DEAE-cellulose			50 mmol/L PBS (pH 6.0), 0-300 mmol/L NaCl			UV-Vis (280 nm)			IEC			[59]		
15	Gypsum- Anemarrhenae composite	metal cations (Na^+^, K^+^, Mg^2+^, Ca^2+^)				IonPac CS12A			20 mmol/L MSA			suppressor CD			IEC			[65]		
16	Rhizoma chuanxiong, Semen zizyphi spinosae	metal cations (Na^+^, N, K^+^, Mg^2+^, Ca^2+^)				ICS-C25			2.5 mmol/L pyromellitic acid			suppressor CD			IEC			[66]		
17	oral preparations		metal cations (Na^+^, K^+^, Mg^2+^)				Dionex^®^ IonPac^®^ CS16			A: 6.7% MSA aqueous solution, B: water			suppressor CD			IEC			[67]		
18	Jinyinhua		metal cations (K^+^, Na^+^, Mg^2+^, Ca^2+^)				IonPac CS12A			20 mmol/L MSA			suppressor CD			IEC			[68]		
19	Melilotus officinalis		inorganic anions (Cl^-^, N, S)				Dionex IonPac AS22			ultrapure water- NaHCO_3_ (1.5 mmol/L)- Na_2_CO_3_ (4.5 mmol/L)			suppressor CD			IEC			[69]		
20	Danshen injection		inorganic anions (F^-^, HCOO^-^, Cl^-^, Br^-^, N, N)				IonPac AS11-HC			12-20 mmol/L KOH solution			suppressor CD			IEC			[70]		
21	Panax notoginseng		inorganic anion (Cl^-^)				IonPac AS15			30 mmol/L KOH solution			suppressor CD			IEC			[71]		
22	Ligusticum chuanxiong		inorganic anions (H_2_P, Cl^-^, N)				Shim-pack IC-A1			potassium hydrogen phthalate solution			direct CD			IEC			[72]		
23	Angelicae sinensis radix		microelements (Sb(V, Ⅲ))				IonPac AS14			50 mmol/L DAP- 50 mmol/L tartaric acid (pH 6.5)			IC-BEcHG-AFS			IEC			[74]		
24	Asini corii colla		microelement (Cr(Ⅵ))				IonPac AS19			30 mmol/L KOH solution			UV (545 nm)			IEC			[75]		
25	Tricholomamatsutake		microelement (As)				Dionex IonPac AS7			A: 2.5 mmol/L (NH_4_)_2_CO_3_, B: 100 mmol/L (NH_4_)_2_CO_3_			IC-ICP-MS			IEC			[76]		
26	Chenxiang Huaqi pill		microelements (As(Ⅲ, V), Cr(Ⅵ))				IonPac AS7, IonPac AG7			5-100 mmol/L (NH_4_)_2_- CO_3_, 0.76 mmol/L NH_4_NO_3_(pH 9.3)			IC-ICP-MS			IEC			[77]		
27	Jasminum sambac, Glycyrrhizae radix et rhizoma, Citri reticulatae pericarpium, etc.		inorganic anions (F^-^, Cl^-^, N, S, P)				self-assembly (YSA-4A model)			water, 4 mmol/L NaHCO_3_-2 mmol/L Na_2_CO_3_ solution			direct CD			IEC			[78]		
28	Coptis herbs		organic acids (quinic acid, acetic acid, formic acid, tartaric acid, malic acid, succinic acid, oxalic acid)				IonPac AS11-HC			1-60 mmol/L KOH solution			suppressor CD			IEC			[83]		
29	Fructus mume herbs		organic acids (lmalic acid, oxalic acid, fumaric acid, citric acid)				Dionex IonPac AS19			4-54 mmol/L KOH solution			suppressor CD			IEC			[84]		
30	Ganoderma lucidum		organic acids (oxalic acid, tartaric acid, malic acid, glycolic acid)				Transgenomic ICSep Ion 300			8 mmol/L H_2_SO_4_ solution			suppressor CD			IEC			[85]		
31	Shengmai injection		organic acids (citric acid, malic acid, succinic acid)				Transgenomic Sep- ION-300			5 mmol/L H_2_SO_4_- acetone (95∶5, v/v)			suppressor CD			ICE			[86]		
32	rice wine		organic acids (13 kinds of oxalic acid, tartaric acid, ascorbic acid, etc.)				IC-Pak Ion Exclusion			H_2_SO_4_-ACN (98∶2, v/v)			UV (210 nm)			ICE			[87]		
33	Ephedrae herba		alkaloid (ephedrine hydrochloride)				Dionex AG9-HC			8 mmol/L Na_2_CO_3_- MeOH (9∶1, v/v)			ECAM (DC amperometric detection)			IEC			[94]		
34	molasses		alkaloid (betaine)				WY-Cation-1			2 mmol/L MSA			direct CD			IEC			[95]		
35	Zuojin pill		alkaloids (13 kinds of berberine hydrochloride, palmatine hydrochloride, evodiamine, rutaecarpine, etc.)				Diamonsil C18			A: 1.5 mmol/L SDS (adjust pH 5.0 with H_3_PO_4_), B: ACN			UV (265 nm)			IPC			[96]		
36	compound Kushen injection			alkaloids (matrine, oxymatrine, sophocarpine, oxysophocarpine)	Diamonsil C18			A: 0.04% H_3_PO_4_- 10 mmol/L sodium pentanesulfonate aqueous solution, B: ACN			UV (210 nm)			IPC			[97]		
37	Uncaria sessilifructus			alkaloid (total alkaloids)	PhenoSphere SCX			A: 20 mmol/L NH_4_OAc- 0.05% formic acid aqueous solution, B: MeOH			UV (254, 203 nm)			IEC			[98]		
38	Atractylodis macrocephalae rhizoma, Dioscoreae rhizoma, Armeniacae semen amarum, Lonicerae flos, etc.			sulfur dioxide residue	IonPac@AS11-HC			20 mmol/L KOH solution			suppressor CD			IEC			[110]		
39	Rhei radix et rhizoma			sulfur dioxide residue	IonPac AS18			NaOH solution			suppressor CD			IEC			[111]		
40	bitter almond, Persicae semen			sulfur dioxide residue	Dionex Ionpac AS11A			12 mmol/L KOH solution			suppressor CD			IEC			[112]		
41	Gastrodiae rhizoma			sulfur dioxide residue	A-SUPP5-150			3.2 mmol/L Na_2_CO_3_ solution, 1 mmol/L NaHCO_3_ solution			suppressor CD			IEC			[113]		
42	chicory roots			sulfur dioxide residue	IonPac AS-11-HC			20 mmol/L KOH solution			suppressor CD			IEC			[114]		

* A, B, E1 and E2: mobile phases of different systems; ACN: acetonitrile; TBAH: tetrabutylammonium hydroxide; CPBS: citric acid buffer solution; CP-BBS: citric acid boric acid buffer solution; EDTA: ethylenediaminetetraacetic acid; DPA: 2,6-pyridinedicarboxylic acid; HFBA: heptafluorobutyric acid; TFA: trifluoroacetic acid; PBS: phosphate buffer; MSA: methanesulfonic acid aqueous solution; DAP: ammonium phosphate dibasic; SDS: sodium dodecyl sulfate. ECAM: electrochemical amperometric method; UV-Vis: ultraviolet-visible absorption spectrophotometry; PAD: pulsed amperometric detection; CD: conductivity detection; BEcHG: bianode electrochemical hydride generation; AFS: atomic fluorescence spectrometry; ICP: inductively coupled plasma; IEC: ion exchange chromatography; IPC: ion pair chromatography; ICE: ion exclusion chromatography; DC: direct current.

## 3 IC(联用)新技术及其在中草药分析中的最新应用进展

IC目前已广泛应用于中草药中无机盐与微量元素、蛋白质与氨基酸和二氧化硫残留等物质的分析。但是,由于中草药中各种代谢物的酸碱性、极性、分子质量与含量跨度较大,单体成分的检测更具挑战性,这对IC(联用)新技术的发展提出了更高要求。由此,本部分重点对现代IC(联用)新技术的研究趋势及其在中草药领域的最新应用进行总结。

### 3.1 IC与其他色谱方法的联用技术及其应用

IC与其他检测技术联用能够极大地提高IC分析的准确性和灵敏度,以扩展其在中草药复杂成分中的应用,下面主要介绍IC-MS、IC-ICP-MS和IEC-HILIC-RPC等3种IC联用技术。首先,IC-MS所用的主要分离方法为离子交换色谱,常与“IEC-MS”一词互为同义词。该方法可用于分离、鉴定和定量复杂组分中广泛的可离子化成分,包括来自无机、有机、环境和生物来源的化合物,目前大多应用于环境科学、法医学、食品科学等领域,但在药物科学、微生物学和代谢组学等领域的研究也日益增长,有进一步发展和应用的空间^[115]^。其次,IC-电感耦合等离子体质谱法是研究复杂基体中痕量元素形态分析的有效手段,已成为As、Pb、Hg等元素分析的优势方法^[116]^。该法具备优于电导或UV检测器的高灵敏度,并可利用IC抑制器实现在线除盐,避免盐在锥口堆积造成堵塞。通过对IC-MS和IC-ICP-MS联用技术的应用方向进行关键词共现性分析(见图3),可以看出目前这两种技术大多数应用于样品中无机盐或微量元素的检测及形态分析,且在中草药领域具备相似应用^[76,77]^。

**图 3:**
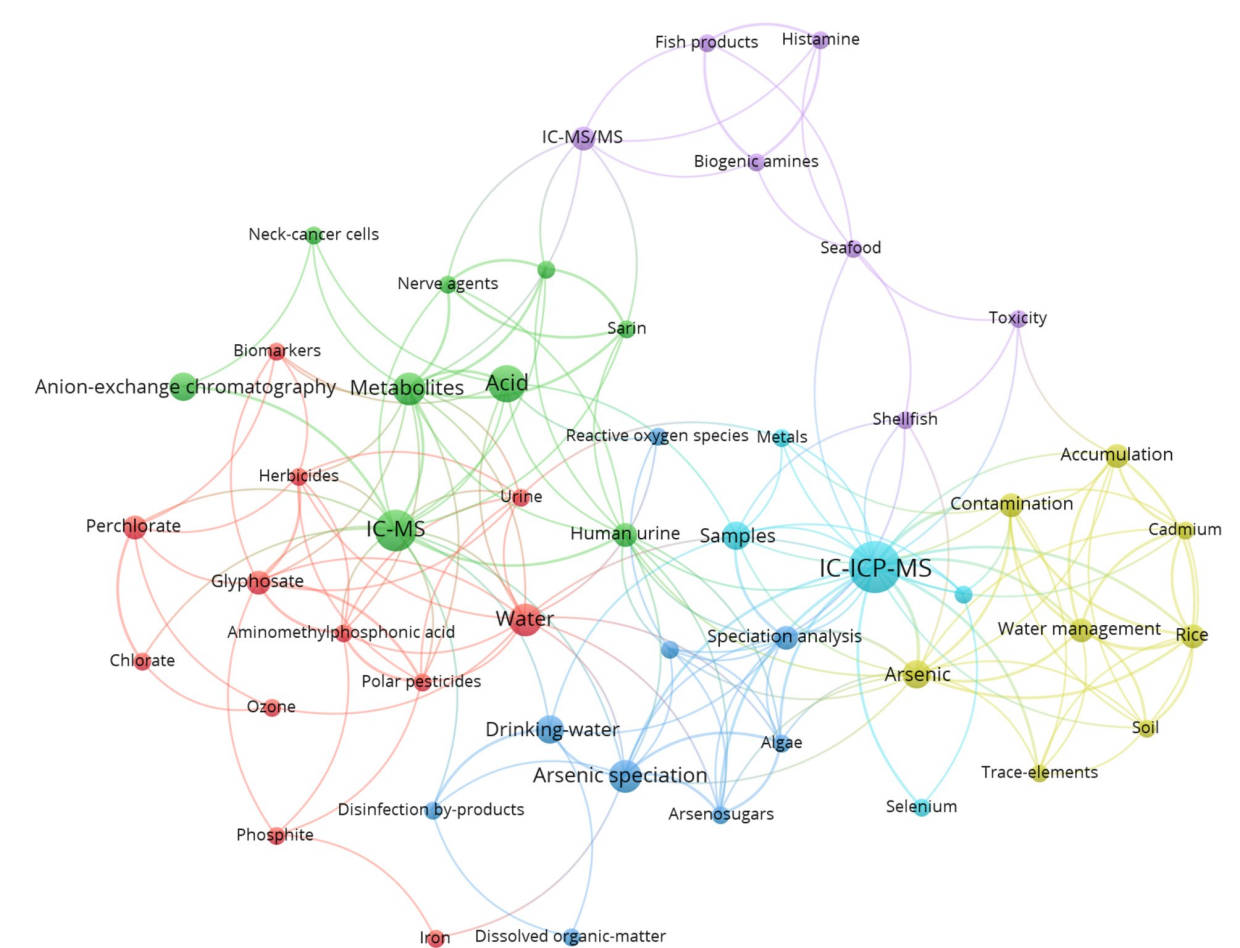
基于IC-MS/IC-ICP-MS研究方向的关键词共现性分析(基于Web of Science检索)

第三种则是Feng等^[98]^在多维色谱(multi-dimensional chromatography, MDC)处理复杂样品时所展现出的突出优势的启发下,首次建立的IEC-HILIC-RPC, 3D-LC色谱系统。他们整合了3种不同的色谱机制,实现了多种化学物质的充分分离,进而从药用植物白钩藤中分离得到308个化合物,鉴定或初步表征了128个化合物,为阐明含有复杂成分的中药材的化学基础提供了解决思路。总之,IC与其他检测方法联用的分析思路已成为研究复杂基体成分的一种有效手段,能够表现出更高水平的稳定性、重现性、灵敏度和低检出限等,未来在中草药领域的应用也将进一步发展。

### 3.2 IC系统部件(在线前处理配置、抑制器、固定相等)的发展及其应用

在线前处理配置是IC中进行样品前处理的重要部分,目的是减少或除去样品中的干扰杂质、调节pH值、降低基体浓度等,使之符合IC仪器的进样要求^[117]^。一直以来,研究者不断改进发展适宜的在线样品前处理方法,林红梅等^[118]^在2012年就利用戴安公司的在线除氯技术,结合OnGuard Ba柱,建立了一种柱前除去氯、硫酸盐的有效方法,解决了过去高盐基体对其他阴离子测定干扰的难题。在中草药领域,Yang等^[75]^建立了一种在线样品前处理的IC系统,首先采用聚合物基反相色谱柱(预处理柱)保留样品中复杂的有机化合物基质,之后通过阀切换技术将带有目标离子的样品溶液输送到分析柱中,此方法可测定阿胶中的痕量Cr(Ⅵ),方便,实用。

抑制器是IC系统的关键部件,当前抑制器经过几十年的改进和发展,通过创新设计和精致配置,解决了过去填充柱抑制器的某些局限性,如延迟体积太大、不具备足够的抑制能力等^[119]^。此前,Yang等^[120]^建立了一种结合电渗膜抑制器与电荷检测器(CHD)的一体化集成装置(命名为Sup-CHD),该装置能够简化系统复杂性,减少可能的柱外分散;它是一种流通式设计,由离子交换膜和离子筛隔离的5个腔室组成,并采用三通道夹层结构,该集成器件具有与单独电渗膜抑制器或CHD类似的功能,但其死体积和分散度比器件单独使用分别降低了18%和37%。在实际应用中,Geerdink等^[121]^还开发了一种简单、稳定、灵敏的IC-MS联用方法,用于中低盐度地表水中草甘膦、草铵磷及其代谢物氨甲基膦酸(AMPA)等的分析,该方法采用一种改良的化学抑制器,其交换膜转化为铵盐形式(由酸性膜转化为碱性膜),这种转换使得AMPA的灵敏度提高了100倍以上。但在检索文献资料时发现,当前尚无通过针对性改变抑制器以适应中药成分分析的研究,可应用于中草药复杂成分的检测中。

固定相(分析柱填料)作为IC系统中的核心部件,它的性质在很大程度上决定了IC的选择性和分离分析效率^[122]^,其制备方法对IC在中草药中的新发展也至关重要。例如Zatirakha等^[123]^整理了莫斯科国立大学分析化学系色谱实验室在IC新型固定相开发方面的研究,该团队成功地改进了以聚合物和无机基质为基础的共价固定吸附剂全新方法,以确保阴离子交换剂的最高选择性和效率,适应更复杂的组分分析。同时,中草药又可作为新型生物固定相填料制备的原料,例如Mojtaba等^[124]^通过对14种中草药药渣的预筛选实验,考察了中药菊苣废弃物及其用CaCl_2_改性后对Pb^2+^和Cd^2+^的离子交换能力,结果显示改性后的菊苣药渣对Pb^2+^和Cd^2+^的最大吸附量分别从103.1 mg/g和53.8 mg/g提高到123.5 mg/g和64.5 mg/g,以菊苣药渣为原料制备生物离子交换剂是一种有前景、低成本替代合成树脂去除废水中重金属的方法。综上,IC的不断发展正推动中草药复杂成分的高效分离分析,同时IC系统的各部件也能够在中草药等生物相关领域的影响下更好发展。

## 4 总结与展望

近年来,IC技术已发展成为中草药化学成分分析的重要方法,同时也越来越被广泛地应用于中药复杂组分的研究。通过对现有文献的检索分析,目前IC在中草药的应用主要包括糖苷类、蛋白质类、有机酸类物质和阴阳离子的测定以及某些金属阳离子的形态分析等,其中最常用的IC类型及检测方式为IEC和电导检测法,与传统的分析方法相比拥有独特优势。但有关IC在无机阴离子的形态分析,黄酮类、苯丙素类和甾体类等主要活性物质中的直接应用研究报道较少。这些活性成分虽大多不属于典型的离子型化合物,不具有明确电荷,但可在特定条件下,由于氢离子化和离子对形成等因素而表现出离子特性,由此利用IC便有可能实现对它们的有效分离分析。此外,类似于将菊苣药渣制备成生物离子交换剂的研究同步促进了IC的更好发展,样品前/后处理方法与检验检测技术两大领域相辅相成、共同发展。

为提升对中草药中具有酸碱性、极性、分子质量与含量跨度大等特点的初级和次级代谢物的检测水平,未来对IC新技术的发展提出了更高要求。展望未来,为适用中草药复杂活性成分的研究,IC技术预计将在以下三方面着力发展:一是样品(在线)前处理技术^[125]^能够使待测物质转化制备为适合IC柱分析的组分,并针对不同组分实现选择性高效富集与分离;二是新型固相材料的创新研究亦可提高IC的分离效果和分析灵敏度,并为其更广泛应用提供支撑;三是IC与质谱等组成的多维色谱联用技术,可以结合各自的优点,为中草药主要活性物质的分析鉴定建立重要方法基础。
